# The chemokine RANTES is secreted by human melanoma cells and is associated with enhanced tumour formation in nude mice

**DOI:** 10.1038/sj.bjc.6690164

**Published:** 1999-03

**Authors:** U Mrowietz, U Schwenk, S Maune, J Bartels, M Küpper, I Fichtner, J-M Schröder, D Schadendorf

**Affiliations:** Department of Dermatology, University of Kiel, Kiel, D-24105, Germany; Department of Oto-Laryngology, Head and Neck Surgery, University of Kiel, Kiel, D-24105, Germany; Max Delbrück Center of Molecular Medicine, Berlin, D-13125, Germany; Clinical Cooperation Unit for Dermatooncology (DKFZ), University of Heidelberg, Mannheim Hospital, Mannheim, D-68135, Germany

**Keywords:** melanoma, metastasis, RANTES, chemokine, human

## Abstract

Modulation of tumour cell growth by tumour-infiltrating leucocytes is of high importance for the biological behaviour of malignant neoplasms. In melanoma, tumour-associated macrophages (TAM) and tumour-infiltrating lymphocytes (TIL) are of particular interest as inhibitors or enhancers of cell growth. Recruitment of leucocytes from the peripheral blood into the tumour site is mediated predominantly by chemotaxins, particularly by the group of chemokines.

The aim of this study was to identify peptides released by human melanoma cells with monocyte chemotactic properties. To assure the presence of biologically active mediators, biochemical purification and biological characterization of peptides was based on a detection system dependent on bioactive, monocyte chemotactic activity in vitro. Cell culture supernatants of melanoma cells were fractioned by heparin–sepharose followed by preparative reversed-phase HPLC steps to enrich monocyte chemotactic activity in one single band on a sodium dodecyl sulphate polyacrylamide gel electrophoresis (SDS-PAGE) gel. These purified fractions were shown to react with RANTES-specific antibodies in an enzyme-linked immunosorbent assay (ELISA) as well as in Western blot analysis. Amino acid sequencing of the N-terminal protein fragment confirmed 100% homology to the RANTES protein. Further analysis showed that four out of eight melanoma cell lines constitutively expressed and secreted the β-chemokine RANTES as detected by ELISA. The amount of RANTES protein secreted (up to 50 ng ml^−1^) was about 5–50 times higher than interleukin 8 (IL-8), determined in the same supernatant samples. Tumour necrosis factor α (TNF-α), not, however, IL-2, interferon-γ (IFN-γ), or α-melanocyte-stimulating hormone (α-MSH) was able to up-regulate RANTES and interleukin 8 secretion. Furthermore, higher levels of RANTES secretion in vitro were associated with increased tumour formation upon S.C. injection of six human melanoma cell lines in nude mice. Our data provide evidence that a subset of melanoma cells express mRNA and secrete RANTES protein which may be partly responsible for the recruitment of monocytes, T-cells and dendritic cells into the tumours. However, transplantation experiments in nude mice suggest that effects of RANTES may also benefit tumour progression. Further studies are needed to dissect the underlying mechanisms. © 1999 Cancer Research Campaign


*British Journal of Cancer* (1999) **79**(7/8), 1025–1031
© 1999 Cancer Research Campaign
Article no. bjoc.1998.0164

### The chemokine RANTES is secreted by human melanoma cells and is associated with enhanced tumour formation in nude mice

**U Mrowietz[1], U Schwenk[1], S Maune[2], J Bartels[1], M Küpper[1], I Fichtner[3], J-M Schröder[1] and D Schadendorf[4]**

[1]Department of Dermatology and [2]Department of Oto-Laryngology, Head and Neck Surgery, University of Kiel, D-24105 Kiel, Germany; [3]Max Delbrück Center of Molecular Medicine, D-13125 Berlin, Germany; [4]Clinical Cooperation Unit for Dermatooncology (DKFZ), University of Heidelberg, Mannheim Hospital, D-68135 Mannheim, Germany

**Summary** Modulation of tumour cell growth by tumour-infiltrating leucocytes is of high importance for the biological behaviour of malignant neoplasms. In melanoma, tumour-associated macrophages (TAM) and tumour-infiltrating lymphocytes (TIL) are of particular interest as inhibitors or enhancers of cell growth. Recruitment of leucocytes from the peripheral blood into the tumour site is mediated predominantly by chemotaxins, particularly by the group of chemokines.

The aim of this study was to identify peptides released by human melanoma cells with monocyte chemotactic properties. To assure the presence of biologically active mediators, biochemical purification and biological characterization of peptides was based on a detection system dependent on bioactive, monocyte chemotactic activity in vitro. Cell culture supernatants of melanoma cells were fractioned by heparin–sepharose followed by preparative reversed-phase HPLC steps to enrich monocyte chemotactic activity in one single band on a sodium dodecyl sulphate polyacrylamide gel electrophoresis (SDS-PAGE) gel. These purified fractions were shown to react with RANTES-specific antibodies in an enzyme-linked immunosorbent assay (ELISA) as well as in Western blot analysis. Amino acid sequencing of the N-terminal protein fragment confirmed 100% homology to the RANTES protein. Further analysis showed that four out of eight melanoma cell lines constitutively expressed and secreted the β-chemokine RANTES as detected by ELISA. The amount of RANTES protein secreted (up to 50 ng $ml^{-1}$) was about 5–50 times higher than interleukin 8 (IL-8), determined in the same supernatant samples. Tumour necrosis factor α (TNF-α), not, however, IL-2, interferon-γ (IFN-γ), or α-melanocyte-stimulating hormone (α-MSH) was able to up-regulate RANTES and interleukin 8 secretion. Furthermore, higher levels of RANTES secretion in vitro were associated with increased tumour formation upon s.c. injection of six human melanoma cell lines in nude mice. Our data provide evidence that a subset of melanoma cells express mRNA and secrete RANTES protein which may be partly responsible for the recruitment of monocytes, T-cells and dendritic cells into the tumours. However, transplantation experiments in nude mice suggest that effects of RANTES may also benefit tumour progression. Further studies are needed to dissect the underlying mechanisms.

**Keywords:** melanoma; metastasis; RANTES; chemokine; human

Tumour-infiltrating leucocytes play a key role in the growth characteristics of malignant neoplasms. In a recent consensus conference discussing histopathology and prognostic factors in malignant melanoma, the importance of the intratumoral infiltrate containing cells of the monocyte/macrophage lineage for the prognosis of this highly aggressive tumour has been particularly emphasized (Barnhill, 1993). Tumour-associated macrophages (TAM) are regularly found in primary lesions as well as in metastasis of melanoma (Bröcker et al, 1988). Quantitative analysis revealed that TAM are the most abundant leucocytes in melanoma (Van Ravenswaay-Claasen et al, 1992). TAM are thought to mediate primarily cytostatic/cytotoxic effects, but may also enhance growth of melanoma cells (Te Velde and Figdor, 1991; Schadendorf et al, 1993). Recruitment of monocytes/macrophages into the tumour seems to be mediated by chemotactic factors generated by tumour cells themselves or, at later stages, also by factors produced by infiltrating leucocytes. In recent years, chemotaxins belonging to the group of chemokines have been found to be profoundly involved not only in inflammatory processes and artherosclerosis, but also in leucocyte recruitment into tumours (Schall and Bacon, 1994). Chemotactic migration of human monocytes has recently been demonstrated to be mediated by β-chemokines such as monocyte chemotactic protein 1 (MCP-1) or macrophage-inflammatory proteins (MIPs) (Uguccioni et al, 1995). Furthermore, melanoma cells have been shown to express mRNA and to secrete MCP-1 protein (Graves et al, 1992). Benomar et al (1987) reported that human melanoma cells with high tumorigenic potential in a nude-mouse model secreted no or low amounts of an unknown monocyte-chemotactic factor. Culture supernatants from melanoma cells with low tumorigenic capacity, however, contained large amounts of that unidentified monocyte-chemotactic factor.

The aim of our study was to identify proteinaceous factors secreted by human melanoma cells that could induce chemotactic migration of highly purified, unstimulated monocytes. To assure the presence of biologically active protein, the first step of our experimental approach consisted of biochemical purification and

*Received 9 February 1998*
*Revised 21 July 1998*
*Accepted 23 July 1998*

*Correspondence to:* U Mrowietz, Department of Dermatology, University of Kiel, Schittenhelmstr. 7, 24105 Kiel, Germany biological characterization of peptides selected according to their chemotactic activity for monocytes in vitro using a chemotaxis bioassay.

### MATERIALS AND METHODS

### Establishment of melanoma cell lines from metastasis

Cell lines from human melanoma metastases from five different patients with stage IV melanoma were established and further cultured. Localization of primary tumours and of metastasis as well as the type of primary melanomas and their tumour thickness are shown in Table 1. All melanoma cell lines used in this study have been thoroughly phenotypically characterized as recently described (Schadendorf et al, 1996).

Briefly, metastases were taken directly after surgery, the surrounding tissue was removed, the metastasis cut into small pieces, and incubated in modified Eagle medium (MEM) (Bio Concept, Umkirch, Germany) with collagenase/dispase (1%, w/v) for 1 h at 37°C. Thereafter, the cells were minced through a nylon mesh, washed, suspended in 'melanoma medium' [MEM supplemented with 10% fetal calf serum (FCS), 2 mM L-glutamine, 100 U $ml^{-1}$ penicillin, 100 mM streptomycin and 0.08 IE $ml^{-1}$ human insulin (Hoechst, Frankfurt, Germany)] and finally seeded into a tissue culture flask. Melanoma cells were grown near confluency in 'melanoma medium'. Cells used for experiments were between passage 4 and 6.

### Culture of melanoma cell lines from primary melanomas

Established cell lines from primary melanomas (WM 115, WM 98-1, WM 1341) were kindly supplied by M Herlyn (The Wistar Institute, Philadelphia, PA, USA) (Balaban et al, 1984; Herlyn et al, 1985; Cornil et al, 1991). Cells were grown in 'melanoma medium' near confluency for the experiments described.

### Treatment of melanoma cell cultures

Melanoma cell cultures were treated by addition of the following mediators or substances: phorbol-myristate acetate (PMA, 1 ng $ml^{-1}$; Sigma, Deisenhofen, Germany), human recombinant interferon-γ (IFN-γ, 100 U $ml^{-1}$; Rentschler, Laupheim, Germany), human recombinant tumour necrosis factor α (TNF-α, 100 U $ml^{-1}$; PeproTech, Rocky Hill, USA), human recombinant interleukin 2 (IL-2, $10^{-8}$ M; PeproTech), α-melanocyte-stimulating hormone (α-MSH, $10^{-8}$ M; Sigma), or 'melanoma medium' alone for 24 or 48 h. Thereafter, supernatants were harvested and stored in aliquots at –80°C until further use. Cells were then washed and subjected to mRNA isolation.

### Biochemical characterization of monocyte chemotactic peptides

Supernatants of unstimulated melanoma cell cultures were first concentrated by ultrafiltration using an Amicon YM-3 membrane. Thereafter, concentrated supernatants were passed over a heparin–sepharose column. Fractions binding to the heparin–sepharose column were tested for monocyte chemotactic activity as follows: heparin-binding proteins were eluted from the column using a continuous gradient of sodium chloride, and the fractions tested for monocyte chemotactic activity. Active fractions were pooled, and further subjected to a preparative C8-reverse-phase high-performance liquid chromatography HPLC column. Fractions were again tested for monocyte chemotactic activity, active fractions were pooled and thereafter analysed using the Smart-HPLC system (Pharmacia) with a C18-reverse-phase column. Fractions with monocyte chemotactic activity were than subjected to sodium dodecyl sulphate polyacrylamide gel electrophoresis (SDS-PAGE).

### Western blot analysis

Fractions showing a single band on SDS-PAGE were subjected to Western blot analysis and were transferred to nitrocellulose in a semidry electrophoresis system (Pharmacia) at pH 8.3, using a Tris (25 mM)/glycine (192 mM) buffer in aqueous methanol (30%, v/v). After blotting, the filters were blocked with 1% (w/v) gelatin in phosphate-buffered saline (PBS), followed by a 1-h incubation with a specific murine monoclonal antibody against RANTES (Sticherling et al, 1995). After twofold washing and incubation with peroxidase-labelled secondary antibody and subsequent washing, the bands were visualized by the use of an enhanced chemiluminescence system kit (Boehringer Mannheim, Mannheim, Germany).

### Amino acid sequence determination

Amino acid sequence was determined by Edman degradation in an Applied Biosystems 476 A pulsed liquid protein sequencer using reversed-phase HPLC for phenylthiohydantoin-derivative detection. Cysteine residues were confirmed after filter reduction with tributylphosphine and alkylation with 4-vinylpyridine.

**Table 1** Origin and clinical characteristics of melanoma cell lines investigated

| Melanoma cell line | Source of metastasis | Gender | Tumour type | Tumour thickness (mm) | Localization of PT | Reference |
|---|---|---|---|---|---|---|
| KI-MEL-7 | Cutaneous | Male | NM | 5.0 | Neck | Schadendorf et al (1996) |
| KI-MEL-13 | Cutaneous | Female | NM | 7.0 | Arm | Schadendorf et al (1996) |
| UKRV-MEL-2 | Pleural effusion | Female | SSM | 1.8 | Arm | Artuc et al (1995) |
| UKRV-MEL-3 | Cutaneous | Female | NM | 2.4 | Knee | Artuc et al (1995) |
| UKRV-MEL-4 | Liver | Female | Ocular | – | Eye | Artuc et al (1995) |
| WM 98-1 | Primary melanoma | n.k. | SSM | n.k. | n.k. | Herlyn et al (1985*a*) |
| WM 1341 | Primary melanoma | n.k. | SSM | n.k. | n.k. | Cornil et al (1991) |
| WM 115 | Primary melanoma | n.k. | NM | n.k. | Right leg | Herlyn et al (1985*b*) |

NM, nodular melanoma; SSM, superficial spreading melanoma; n.k., not known.

*© Cancer Research Campaign 1999*

### Determination of monocyte chemotactic activity using a chemotaxis bioassay

Chemotactic activity for human monocytes in the chromatographic fractions was determined using a chemotaxis bioassay as recently described (Mrowietz and Jürgens, 1995). Briefly, highly purified, unstimulated human monocytes were purified from the peripheral blood of patients after minor cutaneous surgery or from healthy donors, after obtaining informed consent, by Ficoll-paque density centrifugation followed by counterflow centrifugation elutriation (CCE). Viability of monocytes was controlled by trypan-blue dye exclusion, purity was analysed by microscopic evaluation of Giemsa-stained cytospin preparations.

Determination of chemotactic activity was performed using a 48-well chemotaxis chamber (Nuclepore, Bodenheim, Germany) with a PVP-free polycarbonate membrane (pore size 5 µm; Nuclepore). Migrated cells were quantified by densitometry. As a positive control for chemotaxis, *N*-formyl-methionyl-leucyl-phenylalanin (fMLP, $10^{-8}$ M, Sigma) was used in each assay. Random migration of monocytes was controlled using phosphate-buffered saline with 0.1% (w/v) bovine serum albumin, 0.5 mM magnesium chloride and 0.9 mM calcium chloride.

### Determination of RANTES- and IL-8-specific immunoreactivity

Immunoreactivity for human RANTES and IL-8 was tested using sandwich ELISAs, which use specific monoclonal antibodies as described previously (Sticherling et al, 1995, 1989).

### RNA extraction

Cell pellets from cultured melanoma cells were lysed in 1 ml Trizol reagent (Gibco, Eggenstein, Germany) by vigorous pipetting. Total RNA isolation was performed using a modified one-step guanidinium thiocyanate method according to the manufacturer's instructions. Extracted RNA was dissolved in diethylpyrocarbonate (DEPC)-treated water. Integrity and amount of RNA was checked by gel electrophoresis and spectrophotometric analysis.

### Northern blot analysis

Expression of specific mRNA for RANTES was first analysed using a non-radioactive Northern blot technique as previously described (Engler-Blum et al, 1993). Briefly, 10 µg of total RNA was separated by electrophoresis using a 1.2% agarose formaldehyde gel, subsequently transferred to a positively charged nylon membrane and cross-linked by UV light. Hybridization was performed with a RANTES-specific cDNA probe (Pharmacia) labelled with DIG-dUTP (Boehringer). For control, the stripped membrane was rehybridized with mitochondrial 16S ribosomal RNA (Boehringer). Specific binding of DIG-labelled probes was determined by autoradiography of chemiluminescense using Lumigen PPD (Boehringer) as substrate after linking with alkaline-phosphatase-conjugated anti-DIG antibody (Boehringer).

### cDNA synthesis

Five micrograms of total RNA were mixed with 0.5 µg oligo(dT)18 primer (Pharmacia, Freiburg, Germany) in a total volume of 11 µl, incubated for 10 min at 70°C, and subsequently cooled on ice. After equilibration of all reaction components to 42°C, 33.4 mU RNAguard and 0.5 mM dNTP (Pharmacia), 1× first-strand buffer, 10 mM DTT and 200 U superscript II (Gibco) were added to each sample, resulting in a final volume of 20 µl. cDNA synthesis was performed for 50 min at 42°C, followed by enzyme denaturation at 70°C for 15 min. Samples were directly used for polymerage chain reaction (PCR) or stored at –20°C until further processing.

### PCR amplification

PCR was performed in tricine buffer using 1 µM of each specific oligonucleotide primer, 0.2 mM dNTP, 1.25 U *Taq*-polymerase (Gibco) and cDNA equivalent to 1.25 µg RNA in a final volume of 50 µl. To perform 'hot start' PCR, primer and dNTP were adjusted to 30 µl and overlayed with liquid wax (Chill out 14, Biozym, Hess. Oldendorf, Germany) which solidified on ice. The other components of the PCR mixture (20 µl) were added on top of the wax barrier. PCR was started by transferring the samples from ice to the preheated (95°C) thermocycler (Biometra, Göttingen, Germany).

The following primer sequences were used: for GAPDH amplification, a sense (5′-GCT TGT CAT CAA TGG AAA TCC CAT C-3′) and an antisense primer (5′-TGT TGA AGT CAG AGG AGA CCA CCT G-3′) generating a 665-bp fragment was used (Gläser et al, 1997). RANTES was amplified using the sense (5′-CTG CTG CTT TGC CTA CAT TGC-3′) and the antisense primer (5′-CGG GTT CAC GCC ATT CTC CTG 3′) leading to a 545-bp amplificate (Schall et al, 1988; Sticherling et al, 1995).

### Animals and in vivo transplantation

Male NMR/I: nu/nu mice (Bomholtgaard, Ry, Denmark) weighing 20–25 g were age- and weight-matched used. Mice were kept under sterile conditions at 24–26°C room temperature, 50% relative humidity and 12-h light–dark rhythm in laminar flow shelves and were supplied with autoclaved food (Sniff, Soest, Germany) and bedding. The drinking water was filtered and acidified (pH 4.0). For transplantation, melanoma cells were harvested by trypsinization (0.25% trypsin; Seromed, Berlin, Germany) from cell culture flasks and were washed twice with PBS. Subsequently, cells were injected s.c. ($10^7$ cells per mouse) into the right flank of three animals. Mice were visited three times weekly, and growing tumours were measured. Moribund mice or mice whose tumours reached a diameter of 15 mm were killed by cervical dislocation. Metastases were evaluated in all organs by careful macroscopical inspection.

### RESULTS

### Biochemical characterization of monocyte-chemotactic peptides in melanoma cell culture supernatants

The melanoma cell lines KI-MEL-7 and KI-MEL-13 were cultured on a large scale until 5 l of culture supernatants were obtained. Biochemical purification of fractions with monocyte-chemotactic activity in vitro used, sequentially, heparin–sepharose, C8-reversed-phase and C18-reversed-phase HPLC, generating a single band on SDS-PAGE.

In Figure 1, the chromatogram of a preparative RP-8 HPLC is shown. In fractions 10–12, monocyte-chemotactic activity was

© Cancer Research Campaign 1999

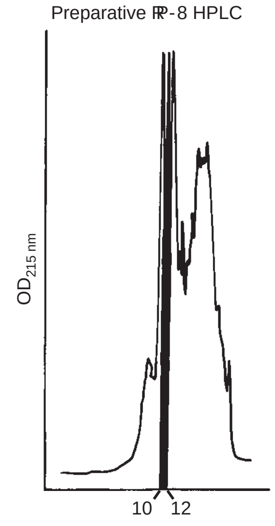

**Figure 1** Profile of a RP-8 HPLC chromatogram of melanoma cell culture supernatant eluted from a heparin–sepharose column. Monocyte-chemotactic activity was detected in fractions 10–12 by chemotaxis bioassay (black area)

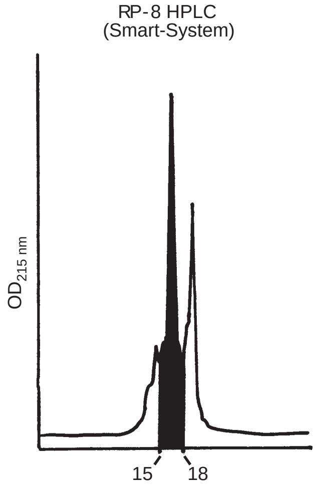

**Figure 2** Purification of melanoma cell-derived monocyte attractant. Micro RP-18-HPLC analysis of chemotactic fractions from RP-HPLC followed by MonoS-cation-exchange HPLC is shown. In fractions 15–18 (black area), strong chemotactic activity for monocytes was detectable detected by chemotaxis assay as well as immunoreactivity for RANTES by ELISA. Further purification by the use of Mono S-cation-exchange HPLC (not shown) followed by micro-RP-18 HPLC on the Smart-System revealed a single peak (fractions 15–18) of chemotactic activity for human monocytes (Figure 2). Peak fractions revealed a single band at 8 kDa upon SDS gel electrophoresis (not shown).

### Determination of immunoreactivity

To test the identity of the monocyte-chemotactic protein, an aliquot of each fraction obtained during the purification procedure was analysed by an ELISA using RANTES-specific monoclonal antibodies. The protein associated with the high monocyte-chemotactic capacity in the chemotaxis bioassay showed strong immunoreactivity by ELISA, implicating that the RANTES protein was isolated and secreted by human melanoma cells. No immunoreactivity was detected when the fraction was tested in an IL-8-specific ELISA (data not shown).

### Western blot analysis

The fraction showing monocyte chemotactic activity together with positive immunoreactivity with the RANTES-specific antibody in ELISA was further analysed by Western blot. A single immuno-reactive band was detectable at 8 kDa for RANTES as control as well as for the purified monocyte attractant, but not, however, for a fraction lacking monocyte chemotactic activity (Figure 3).

### Amino acid determination

To prove that RANTES protein was responsible for the observed effects outlined above, amino acid sequencing was performed. The analysis revealed the following N-terminal amino acid sequence: ser–pro–tyr–ser–ser–asm–tyr–tyr–pro.

This sequence is identical to the amino acid sequence deduced from cDNA of human RANTES (Schall et al, 1988).

We can, therefore, conclude that the protein secreted from human melanoma cells showing monocyte chemotactic activity as well as immunoreactivity with a RANTES-specific antibody is indeed identical to human RANTES.

### Northern blot analysis

Next, we investigated whether human melanoma cell lines express mRNA for RANTES using Northern blot analysis. As shown in Figure 4, the melanoma cell lines KI-MEL-7, KI-MEL-13 and UKRV-MEL-3 demonstrated constitutive expression of significant levels of RANTES mRNA. Addition of PMA did not increase the level of expression. WM 98-1 showed only a weak expression, whereas the melanoma cell lines UKRV-MEL-2 and -4, WM 115 and WM 1341 were found to lack expression of RANTES-specific

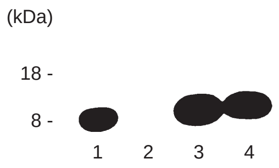

**Figure 3** Identification of RANTES as monocyte attractant. RANTES Western blot analysis was performed with fraction 17 (lane 3), fraction 18 (lane 4), fraction 12 (lane 2) and 01 ng authentic RANTES (lane 1). Note the presence of a single band in fractions 17 and 18 at the same position as authentic RANTES

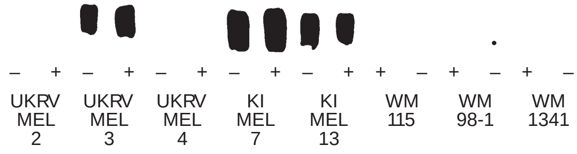

**Figure 4** Expression of RANTES mRNA in cell lines from human melanoma metastasis (UKRV-MEL-2, -3, and -4, KI-MEL-7, and -13), as well as from primary melanomas (WM 115, WM 98–1, and WM 1341) by Northern blot analysis. (–), Without stimulation; (+), after stimulation with PMA (10 ng ml$^{-1}$, 16 h)

 *© Cancer Research Campaign 1999*

**Table 2** RANTES immunoreactivity (ng ml$^{-1}$) of melanoma cell cultures with or without PMA stimulation

| | PMA | RANTES-IR[a] | IL-8-IR[a] |
|---|---|---|---|
| KI-MEL-7 | – | 50 | 1.6 |
| | + | 40 | 1.6 |
| KI-MEL-13 | – | 21.5 | <0.1 |
| | + | 21.5 | <0.1 |
| UKRV-MEL-2 | – | <0.1 | 25 |
| | + | <0.1 | 25 |
| UKRV-MEL-3 | – | 18 | n.d. |
| | + | 24.5 | n.d. |
| UKRV-MEL-4 | – | <0.1 | <0.1 |
| | + | <0.1 | <0.1 |
| WM 98-1 | – | 5.1 | 1.3 |
| | + | 3.9 | 1.8 |
| WM 115 | – | <0.1 | n.d. |
| | + | <0.1 | n.d. |
| WM 1341 | – | <0.1 | 0.4 |
| | + | <0.1 | 0.4 |

[a]RANTES/IL-8 immunoreactivity (ng ml$^{-1}$; ELISA). +, With PMA stimulation (1 ng ml$^{-1}$, 16 h); –, without PMA stimulation. <0.1 = below the detection limit of ELISA. n.d., not done.

mRNA.

### Modulation of mRNA expression for RANTES as determined by PCR

To investigate the influence of certain mediators on RANTES-specific mRNA expression, KI-MEL-13 melanoma cells were incubated with TNF-α, IL-2, IFN-γ, α-MSH or medium alone for 24 or 48 h. After incubation, mRNA was extracted, reverse transcribed and expression of RANTES mRNA analysed by RT-PCR as described in the Materials and methods section. No significant effect of the mediators tested on RANTES mRNA expression was detectable (data not shown).

### Regulation of RANTES and IL-8 protein secretion

To test the inducibility of RANTES protein secretion, five of the newly established cell lines derived from melanoma metastasis and three long-term cell lines from primary melanomas (Table 1) were subjected to PMA treatment (1 ng ml$^{-1}$ for 16 h). Supernatants were analysed for RANTES and IL-8 immunoreactivity. The results are summarized in Table 2, indicating that four out of eight melanoma cell lines (KI-MEL-7, KI-MEL-13, UKRV-MEL-3 and WM 98-1 secreted constitutively RANTES protein. Addition of PMA did not exert any effect on RANTES secretion. The remaining cell lines did not express RANTES protein and could not be induced to do so by addition of PMA. IL-8 immunoreactivity was detected in four out of six melanoma cell lines tested. Only in the supernatants of two of the cell lines, KI-MEL-7 and WM 98-1, was immunoreactivity for both RANTES and IL-8 measured. In both cell lines, RANTES immunoreactivity was found to be two- to 25-fold higher than IL-8.

In Table 3, RANTES as well as IL-8 secretion after stimulation of melanoma cells with IFN-γ, TNF-α, IL-2, α-MSH, or medium alone after 24 or 48 h is shown. Determination of immunoreactivity for RANTES and IL-8 by specific ELISA revealed that only TNF-α was able to up-regulate RANTES protein in KI-MEL-7 and -13 melanoma cells, but not however in the RANTES-negative cell lines UKRV-MEL-2 and -4. IL-8-immunoreactivity could be up-regulated in three out of four melanoma cell lines. In UKRV-MEL-4 cells, IL-8 immunoreactivity was up-regulated by α-MSH. Stimulation of the cell lines investigated for 48 h led to a more pronounced secretion of RANTES and IL-8 immunoreactivity compared with the incubation for 24 h.

**Table 3** Secretion of RANTES and IL-8 protein from human melanoma cells after stimulation with IFN-γ (100 U ml$^{-1}$), TNF-α (100 U ml$^{-1}$), IL-2 ($10^{-8}$ M), α-MSH ($10^{-8}$ M) or medium alone for 24 or 48 h. Immunoreactivity for RANTES and IL-8 was determined by specific ELISA. Mean of three independent experiments

| | RANTES-IR[a] | | IL-8-IR[a] | |
|---|---|---|---|---|
| | 24 h | 48 h | 24 h | 48 h |
| KI-MEL-7 | | | | |
| IFN-γ | 117 | 240 | 33 | 44 |
| TNF-α | 200 | 480 | 35 | 84 |
| IL-2 | 77 | 177 | 14 | 24 |
| α-MSH | 98 | 189 | 37 | 30 |
| Medium | 180 | 218 | 20 | 47 |
| KI-MEL-13 | | | | |
| IFN-γ | 95 | 148 | 6.8 | 7.7 |
| TNF-α | 140 | 173 | 28.5 | 34 |
| IL-2 | 57 | 107 | 4.9 | 9 |
| α-MSH | 67 | 107 | 4.7 | 7.8 |
| Medium | 72 | 142.5 | 4.5 | 7.2 |
| UKRV-MEL-2 | | | | |
| IFN-γ | <0.1 | <0.1 | 7 | 7.7 |
| TNF-α | <0.1 | <0.1 | 28.5 | 34 |
| IL-2 | <0.1 | <0.1 | 4.9 | 9 |
| α-MSH | <0.1 | <0.1 | 4.7 | 7.8 |
| Medium | <0.1 | <0.1 | 7.8 | 7.25 |
| UKRV-MEL-4 | | | | |
| IFN-γ | <0.1 | <0.1 | 3.4 | 5.5 |
| TNF-α | <0.1 | <0.1 | 3 | 6.9 |
| IL-2 | <0.1 | <0.1 | 5 | 4.6 |
| α-MSH | <0.1 | <0.1 | 16.8 | 13.4 |
| Medium | <0.1 | <0.1 | 1.5 | 2.6 |

[a]RANTES/IL-8 immunoreactivity (ng ml$^{-1}$). <0.1 = below the detection limit of ELISA.

### Correlation of RANTES secretion and growth in nude mice

Human melanoma cell lines administered at a fixed cell number into the right flank of mice varied significantly in their growth kinetics (Table 4). KI-MEL-7 and KI-MEL-13 produced massive cutaneous tumours in all mice in a short period (mean 34 days) after injection. Both cell lines are characterized by higher levels of RANTES secreted into culture medium in vitro. Injection of WM 98.1 cells that released medium/low concentrations of RANTES (5.1 ng ml$^{-1}$) in vitro generated reproducible small cutaneous tumours in all animals in 40 days, however no distant metastases were detectable. Cell lines WM1341, UKRV-MEL-2 and UKRV-MEL-4, which did not secrete RANTES, generated very small cutaneous tumours. These tumours, however, stopped growing and mice were still alive after 100 days and metastases could not be found in any organ.

© Cancer Research Campaign 1999

**Table 4** Tumour growth characteristics and development of metastases after s.c. administration of $10^7$ cells into the flank. Three mice were injected in each group. RANTES secretion was determined by ELISA in cell culture supernatant of $10^6$ cells for 24 h. Mean of three experiments

| | RANTES (ng ml$^{-1}$) | Tumour take and metastases |
|---|---|---|
| KI-MEL-7 | 50.0 | Massive cutaneous tumours (3/3) and no metastases after 34 days |
| KI-MEL-13 | 21.5 | Massive cutaneous tumours (3/3) after 34 days and liver metastases with ascites (1/3) after 100 days |
| WM 98.1 | 5.1 | 3/3 cutaneous tumours, mice alive, no metastases |
| UKRV-MEL-2 | <1.0 | 3/3 small cutaneous tumours, mice alive after 100 days; no metastases |
| UKRV-MEL-4 | <1.0 | 2/3 small cutaneous tumours, mice alive after 100 days; no metastases |
| WM 1341 | <1.0 | 3/3 small cutaneous tumours, mice alive after 100 days; no metastases |

### DISCUSSION

Melanoma is a highly malignant tumour derived from melanocytes, with a steadily growing incidence worldwide (Rigel et al, 1996; Rivers, 1996). The tumour tends to develop early metastases mainly via the blood and the lymphatics and is then associated with a poor prognosis. Because results obtained by conventional chemotherapy are highly unsatisfactory and have only palliative effects, in recent years therapeutic attempts have been made to use immunostimulating compounds such as interleukin 2 or interferons for treatment of metastatic melanoma (Ferrone, 1994). Immunostimulating therapeutic approaches are primarily based on the migration to or the presence of leucocytes within the tumour tissue. Tumour-associated macrophages (TAM) can regularly be found in primary melanoma and melanoma metastasis and are, quantitatively, the dominating leucocyte type (Bröcker et al, 1988; Van Ravenswaay-Claasen et al, 1992).

Analogous to inflammatory processes, the recruitment of monocytes from the peripheral blood into tumours such as melanomas is thought to be mediated mainly by chemotaxins. After recruitment, monocytes may differentiate into macrophages or dendritic cells, depending on microenvironmental factors (Ibrahim et al, 1995).

In this study, we aimed at the identification and characterization of monocyte-chemotactic factors secreted by human melanoma cells. For this purpose, five cell lines derived from human melanoma metastasis were freshly established and thoroughly characterized and compared with three established cell lines derived from primary melanomas. Monocyte chemotactic activity could be demonstrated in cell culture supernatants of human melanoma cells and was further subjected to a fractionated biochemical purification scheme and subsequent biological characterization.

The results of our study demonstrated that the β-chemokine RANTES was present in large amounts in pooled culture supernatants of melanoma cells, as finally proven by amino acid sequence analysis. In comparison with the secretion of the α-chemokine IL-8, RANTES secretion was up to 50-times higher.

Further analysis demonstrated RANTES expression and secretion in four out of eight lines tested without stimulation. IL-8 immunoreactivity could be detected in the supernatants of four out of six melanoma cell lines investigated, which is in accordance with previous findings (Schadendorf et al, 1993). In three out of four melanoma cell lines, only TNF-α was able to up-regulate IL-8 secretion in a time-dependent fashion. These data are in accordance with a recent publication showing increased IL-8 mRNA expression and protein secretion in melanoma cells by IL-1β and TNF-α (Singh and Varney, 1998).

Chemokines represent a novel group of proteins with structural similarities and chemotactic activity for certain leucocytic target cells. For monocytes, β-chemokines (C–C chemokines) were shown to be highly potent inducers of chemotactic migration. It has been demonstrated previously by in situ hybridization and immunostaining that melanoma cells are capable of expressing MCP-1 mRNA and a protein which is a prominent β-chemokine (Graves et al, 1992). In a further PCR-screening study, expression of RANTES mRNA was detected in 18 out of 21 melanoma cell cultures, however five human melanocyte cultures were negative (Mattil et al, 1994).

Until recently, chemotactic factors secreted from melanoma cells had not been identified. A supposedly proteinaceous factor with monocyte-chemotactic activity released from human melanoma cells has, however, been described in 1983 by Bottazzi et al (Bottazzi et al, 1983). Secretion of RANTES by the A375 melanoma cell line after lymphotoxin β cross-linking has been recently reported (Degli-Esposti et al, 1997).

The expression and secretion of RANTES by patient-derived melanoma cells and its modulation, as shown in the present study, has not been demonstrated so far. In addition, we have analysed six human melanoma cell lines for their growth kinetics in a nude mouse model after s.c. injection of a fixed number of tumour cells. Cutaneous tumour formation and capacity to develop distant metastases seemed to correlate well with the levels of RANTES released in vitro, but not however with IL-8 secretion. These results point towards distinct differences in both chemokines with regard to the metastatic potential (triggered by RANTES) and growth characteristics (triggered by IL-8) of melanoma cells. In contrast, Benomar et al 1987) reported the correlation of low tumorigenic potential in a nude mouse model and the high expression of an unknown monocyte chemotactic factor released from melanoma cells. Because a large number of chemokines have been identified so far, further studies are needed to elucidate the role of these different compounds as well as the underlying mechanisms.

RANTES does not specifically attract monocytes, but also attracts T-cells of the memory subset (Schall et al, 1990). In addition, Sozzani et al (1995) showed that monocyte-derived dendritic cells respond chemotactically to RANTES, but not however to MCP-1. The detection of RANTES protein in melanoma cell supernatants may, therefore, be of importance for the recruitment not only of monocytes but also of T-cells and dendritic cells into the tumour tissue. The use of nude mice, however, is not a suitable model to study the influence of T-cells and exact immunological interactions.

Taken together, our data show that a subset of human melanoma cells are constitutively capable of expressing mRNA and secreting protein of the β-chemokine RANTES. Compared with other chemokines such as IL-8, the amount of RANTES secretion is

 © Cancer Research Campaign 1999

high. In melanoma cells expressing RANTES mRNA, this chemokine represents the major monocyte-chemotactic activity present in culture supernatants. RANTES may, therefore, be a possible candidate for attracting monocytes from the peripheral blood into the tumour tissue. However, other factors such as newly identified members of the still growing family of chemokines need also to be considered. The growth advantage of RANTES-secreting cell lines might be a first hint in that direction. Further investigations related to the exact role of RANTES in vivo and its influence on the tumorigenicity of melanoma cells in nude mice are needed.

### ACKNOWLEDGEMENTS

The excellent technical assistance of Mrs Anja Ziegenhagen is gratefully acknowleged. RANTES and GAPDH primer were designed and kindly provided by Dr Reinhard Kulke and Dr Regine Gläser, Department of Dermatology, University of Kiel. This work was supported by grants from the Dr Mildred Scheel-Stiftung für Krebsforschung/Deutsche Krebshilfe (W29/92/Mr1), by the Werner und Klara Kreitz-Stiftung für Krebsforschung (both to UM) and by the DFG (Scha 422/5-1; Scha422/6-1) to DS.

### REFERENCES

Artuc M, Nürnberg W, Czarnetzki BM and Schadendorf D (1995) Characterization of gene regulatory elements for selective gene expression in human melanoma cells. *Biochem Biophys Res Commun* **213**: 699–705

Balaban G, Herlyn M, Guerry D4, Bartolo R, et al (1984) Cytogenetics of human malignant melanoma and premalignant lesions. *Cancer Genet Cytogenet* **11**: 429–439

Barnhill RL (1993) Pathology and prognostic factors. *Curr Opin Oncol* **5**: 364–376

Benomar A, Ming WJ, Taraboletti G, Ghezzi P, et al (1987) Chemotactic factor and P15E-related chemotaxis inhibitor in human melanoma cell lines with different macrophage content and tumorigenicity in nude mice. *J Immunol* **138**: 2372–2379

Bottazzi B, Polentarutti N, Acero R, Balsari A, et al (1983) Regulation of the macrophage content of neoplasms by chemoattractants. *Science* **220**: 210–212

Bröcker EB, Zwadlo G, Holzmann B, Macher E, et al (1988) Inflammatory cell infiltrates in human melanoma at different stages of tumor progression. *Int J Cancer* **41**: 562–567

Cornil I, Theodorescu D, Man S, Herlyn M, et al (1991) Fibroblast cell interactions with human melanoma cells affect tumor cell growth as a function of tumor progression. *Proc Natl Acad Sci USA* **88**: 6028–6032

Degli-Esposti MA, Davis-Smith T, Din WS, Smolak PJ, et al (1997) Activation of the lymphotoxin β receptor by cross-linking induces chemokine production and growth arrest in A375 melanoma cells. *J Immunol* **158**: 1756–1762

Engler-Blum G, Meier M, Frank J and Müller H (1993) Reduction of background problems in nonradioactive northern and southern blot analyses enables higher sensitivity than $^{32}$P-based hybridizations. *Anal Biochem* **210**: 235–239.

Ferrone S (1994) Melanoma, immune surveillance, and immunotherapy. *J Clin Invest* **93**: 1351–1352

Gläser R, Rass K, Seiter S, Hauschild A, et al (1997) Detection of circulating melanoma cells by specific amplification of tyrosinase complentary DNA is not a reliable tumor marker in melanoma patients: a clinical two-center study. *J Clin Oncol* **15**: 2818–2825

Graves DT, Barnhill R, Galanopoulos T and Antoniades HN (1992) Expression of monocyte chemotactic protein-1 in human melanoma in vivo. *Am J Pathol* **140**: 9–14

Herlyn M, Balaban G, Bennicelli J, Guerry D4, et al (1985*a*) Primary melanoma cells of the vertical growth phase: similarities to metastatic cells. *J Natl Cancer Inst* **74**: 283–289

Herlyn M, Thurin J, Balaban G, Bennicelli JL, et al (1985*b*) Characteristics of cultured human melanocytes isolated from different stages of tumor progression. *Cancer Res* **45**: 5670–5676

Ibrahim MA, Chain BM and Katz DR (1995) The injured cell: the role of the dendritic cell system as a sentinel receptor pathway. *Immunol Today* **16**: 181–186.

Mattei S, Colombo MP, Melani C, Silvani A, et al (1994) Expression of cytokine/growth factors and their receptors in human melanoma and melanocytes. *Int J Cancer* **56**: 853–857

Mrowietz U and Jürgens G (1995) A method for the determination of leukocyte migration for large sample numbers by automated densitometric quantification. *J Biochem Biophys Methods* **30**: 49–58

Rigel DS, Friedman RJ and Kopf AW (1996) The incidence of malignant melanoma in the United States: issues as we approach the 21st century. *J Am Acad Dermatol* **34**: 839–847

Rivers JK (1996) Melanoma. *Lancet* **347**: 803–806

Schadendorf D, Möller A, Algermissen B, Worm M, et al (1993) IL-8 produced by human malignant melanoma cells in vitro is an essential autocrine growth factor. *J Immunol* **151**: 2667–2675

Schadendorf D, Fichtner I, Makki A, Alijagic S, et al (1996) Metastatic potential of human melanoma cells in nude mice – characterization of phenotype, cytokine secretion and tumour-associated antigens. *Br J Cancer* **74**: 194–199.

Schall TJ, Jongstra J, Dyer BJ, Jorgensen J, et al (1988) A human T cell-specific molecule is a member of a new gene family. *J Immunol* **141**: 1018–1025.

Schall TJ, Bacon K, Toy KJ and Goeddel DV (1990) Selective attraction of monocytes and T lymphocytes of the memory phenotype by cytokine RANTES. *Nature* **347**: 669–671.

Schall TJ and Bacon KB (1994) Chemokines, leukocyte trafficking, and inflammation. *Curr Opin Immunol* **6**: 865–873

Singh RK and Varney ML (1998) Regulation of interleukin 8 expression in human malignant melanoma cells. *Cancer Res* **58**: 1532–1537

Sozzani S, Sallusto F, Luini W, Zhou D, et al (1995) Migration of dendritic cells in response to formyl peptides, C5a, and a distinct set of chemokines. *J Immunol* **155**: 3292–3295

Sticherling M, Schröder JM and Christophers E (1989) Production and characterization of monoclonal antibodies against the novel neutrophil activating peptide NAP/IL-8. *J Immunol* **143**: 1628–1634

Sticherling M, Küpper M, Koltrowitz F, Bornscheuer E, et al (1995) Detection of the chemokine RANTES in cytokine-stimulated human dermal fibroblasts. *J Invest Dermatol* **105**: 585–591

Te Velde AA and Figdor CG (1991) Monocyte-mediated cytotoxic activity against melanoma. *Melanoma Res* **1**: 303–309

Uguccioni M, D'Apuzzo M, Loetscher M, Dewald B, et al (1995) Actions of the chemotactic cytokines MCP-1, MCP-2, MCP-3, RANTES, MIP-1α and MIP-1β on human monocytes. *Eur J Immunol* **25**: 64–68

Van Ravenswaay-Claasen HH, Kluin PM and Fleuren GJ (1992) Tumor infiltrating cells in human cancer. On the possible role of CD16$^{+}$ macrophages in antitumor cytotoxicity. *Lab Invest* **67**: 166–174

© Cancer Research Campaign 1999 
